# Specific high-resolution scheme to improve understanding of the spatio-temporal dispersion of lymphogranuloma venereum epidemic

**DOI:** 10.3389/fmicb.2022.1056216

**Published:** 2022-12-20

**Authors:** Laura Martínez-García, José María González-Alba, Teresa Puerta, Alicia Comunión, María Concepción Rodríguez-Jiménez, Eva Orviz, Matilde Sánchez-Conde, Mario Rodríguez-Domínguez, Rafael Cantón, Juan Carlos Galán

**Affiliations:** ^1^Servicio de Microbiología, Hospital Universitario Ramón y Cajal, Instituto Ramón y Cajal de Investigación Sanitaria (IRYCIS), Madrid, Spain; ^2^Centro de Investigación Biomédica en Red en Epidemiología y Salud Pública (CIBERESP), Madrid, Spain; ^3^Servicio de Microbiología, Hospital Universitario Central de Asturias, Instituto de Investigación Sanitaria del Principado de Asturias (ISPA), Oviedo, Spain; ^4^Centro Sanitario Sandoval, Hospital Clínico San Carlos, Instituto de Investigación Sanitaria San Carlos (IdISSC), Madrid, Spain; ^5^Centro Montesa, Madrid, Spain; ^6^Servicio de Enfermedades Infecciosas. Hospital Universitario Ramón y Cajal, Instituto Ramón y Cajal de Investigación Sanitaria (IRYCIS), Madrid, Spain; ^7^Centro de Investigación Biomédica en Red de Enfermedades Infecciosas (CIBERINFEC), Madrid, Spain

**Keywords:** *Chlamydia trachomatis*, lymphogranuloma venereum, MLST, surveillance, molecular epidemiology

## Abstract

**Introduction:**

Lymphogranuloma venereum (LGV) is already endemic in vulnerable populations in several European countries; however, molecular epidemiology data with improved accuracy are necessary to better understand LGV epidemic in these countries. Current strategies to study the molecular epidemiology of LGV cases involve schemes based on a few genetic fragments of *Chlamydia trachomatis*, which have demonstrated limited discriminatory power for LGV. Therefore, this study aimed to propose a new combination of molecular markers based on the most variable genes of L-genotype genomes to improve the characterization of the current LGV epidemic in Madrid, Spain.

**Methods:**

Four genes were selected according to their diversity index (CTLon_0054, CTLon_0087, CTLon_0243 and CTLon_0301) for use in combination with *ompA*. *In silico* and experimental studies were performed to compare the previously described multilocus sequence typing (MLST) schemes with our proposal. Moreover, the proposed scheme was applied (*n* = 68) to analyze the spatio-temporal spread of the LGV cases.

**Results:**

Our proposal demonstrated higher diversity allowing the identification of three main groups compared to the previously published MLST based on hypervariable genes wherein only a single sequence type was identified. The temporal analysis showed that the major cluster was progressively diversifying, revealing a very active transmission chain. Furthermore, an L2b genome identical to that of the origin of the epidemic was detected, suggesting reintroductions or a low screening rate in vulnerable populations. The spatial distribution suggests that the selection and spread of new variants occurs from the central district to the peripheral regions.

**Discussion:**

The scheme proposed in this study has proven to be useful for appropriate discrimination of LGV strains. This study, to our knowledge for the first time, demonstrates a spatio-temporal spread that increases our understanding and identifies areas with special susceptibility for maintenance of the endemic situation of LGV.

## Introduction

*Chlamydia trachomatis* (CT) is the most common bacterial sexually transmitted infection (STI), causing numerous new infections every year (World Health Organization, 2018). The broad spectrum of diseases caused by CT is related to different genotypes with varying rates off invasiveness. L-genotypes (L1, L2, and L3) are responsible for lymphogranuloma venereum (LGV), whose clinical presentation is associated with inguinal an femoral lymphadenopathies, proctitis, proctocolitis and ulcers, among others (Ceovic and Jerkovic Gulin, 2015; Stoner and Cohen, 2015). Traditionally, LGV was an endemic in tropical areas, but LGV is already currently endemic in several European countries and cases have also been reported in Australia and North America (Hal et al., 2007; Totten et al., 2015; Donachie et al., 2018), mainly associated to men who have sex with men (MSM). In countries with well-established surveillance programs, the incidence is increasing (Marangoni et al., 2021); however, the true extent of the LGV epidemic is not well known. This is due to the atypical clinical presentations, whereby a high proportion are asymptomatic infections (van Aar et al., 2020). In addition, the limited surveillance and notification to public health authorities across the different countries may give rise to a substantial under diagnosis (Cole et al., 2020). Furthermore, data on accurate molecular epidemiology is scarce. These problems contribute to the silent spread of this microorganism (Martínez-García et al., 2021). Therefore, improved screening (Rodríguez-Domínguez et al., 2022) and discovery of new genetic markers, which can be easily implemented in molecular surveillance, could help better understand the LGV epidemic (Borges et al., 2021).

The most common strategy for studying the molecular epidemiology of the LGV epidemic is based on *ompA* (Dean et al., 1991) and *ompA-pmpH* (Rodríguez-Domínguez et al., 2014) sequence analysis. Studies implementing multilocus sequence typing (MLST; Wang et al., 2011) or multilocus variable number tandem-repeat analysis (MLVA) schemes (Pedersen et al., 2008; Bom et al., 2011), which increase the resolution in CT, have been reported; however, this schemes had limited success in LGV due to the low discriminatory power. This could possibly result from the low level of diversity in L-genotypes, which is considerably lower than the genetic diversity in CT, whose diversity is also low (Harris et al., 2012; Patiño et al., 2018). Studies based on whole-genome sequencing (WGS) were published at the beginning of the LGV epidemic (Thomson et al., 2008). This could probably be because this strategy is still expensive and difficult to implement in the laboratory. Therefore, new methods are needed to improve LGV surveillance through the design of an MLST scheme based on the most variable genes of L-genotypes. This new approach should provide a precise view of the dynamic spread of LGV cases because of its expected higher resolution compared to previous strategies. This study aims to propose a new combination of molecular markers that allow the identification of short-time scale evolutionary changes and to perform a spatio-temporal distribution to improve the characterization of the current LGV epidemic in Madrid to help in defining Public Health measures.

## Materials and methods

### Dataset and genes selection

All available complete genome sequences of the L-genotypes of CT (n = 29) were downloaded from the NCBI database.^1^ Accession numbers for the genomes used are: NC_010280; NC_010287; NC_015744; NC_020929; NC_020930; NC_020931; NC_020933; NC_020934; NC_020935; NC_020936; NC_020937; NC_020938; NC_020945; NC_020973; NC_020974; NC_020975; NC_020976; NC_020977; NC_020978; NC_021050; NC_021052; NZ_ACUI01000001; NZ_CP009923; NZ_CP009925; NZ_CP019385; NZ_CP019386; NZ_CP019387; NZ_CVNC01000001; NZ_CVND01000001. The last update of the dataset was in January 2019.

We used the Basic Local Alignment Search Tool (BLAST) and identified 878 genes with AM884177 (L2b/UCH-1) as reference. The sequences were aligned using the multiple alignment fast fourier transform (MAFFT). The mean evolutionary diversity (D), defined as the number of base substitutions per site from the average of the entire population, was estimated for each gene using the Tamura-Nei model (Tamura and Nei, 1993). Evolutionary analyses were conducted using MEGA 7. For genes with greater diversity in L-genotypes, the same analysis was repeated for the L2b lineage as it was the only lineage with more than two sequences available. Genes with the highest D index were selected for the new scheme.

We also studied the genes used in two previously described MLST schemes (Klint et al., 2007; Dean et al., 2009) and the MLVA scheme (Pedersen et al., 2008), using an *in silico* approach to infer the discrimination power of these schemes for L-genotypes. The scheme proposed by Dean et al. (2009) is a CT MLST scheme that includes *glyA*, *mdhC*, *pdhA*, *yhbG*, *pykF*, *lysS*, and *leuS* genes; the MLST scheme proposed by Klint et al. (2007) contains CT046 (*hctB*), CT058, CT144, CT172, and CT682 (*pbpB*) regions, which are considered hypervariable genes, and MLVA scheme includes the variable number tandem repeats of loci CT1291, CT1299 and CT1335 together with *ompA* (Pedersen et al., 2008).

### Sample selection, amplification, and sequencing of proposed genes

The study was carried out using 68 clinical samples obtained from July 2017 to September 2018 from 68 patients who visited the Hospital Universitario Ramón y Cajal and an STI center attending mainly MSM population (CS Sandoval) in whom L-genotypes had been previously detected. Most of the samples were rectal swabs (59/68), followed by 7/68 ulcers, 1/68 adenopathy, and 1/68 cervical samples. The L-genotypes detection was performed using a real time PCR based on 36 bp deletion in *pmpH* gene that was confirmed by *pmpH* sequencing (Rodríguez-Domínguez et al., 2014). This study was approved by the Ethics Committee of the Hospital Universitario Ramón y Cajal (reference no. 012/17).

In the first round, fragments of the selected genes (CTLon_0054, CTLon_0087, CTLon_0243 and CTLon_0301) together with the corresponding genes to MLST of Klint et al. (2007), were amplified and sequenced in 28/68 samples to compare the discrimination power between these strategies. In the second round, only the selected genes were sequenced from the remaining samples (40/68). Moreover, a 990 bp fragment of *ompA*, encoding the major outer membrane protein (MOMP), was also sequenced in all samples. The primers used for the amplification of each proposed gene and *ompA* are shown in Table 1, and the PCR conditions are described in the Supplementary material. Amplification and sequencing of the genes included in the MLST scheme described by Klint et al. (2007) were performed using the previously published conditions. Sanger sequencing was performed using a 3130 Genetic Analyzer (Applied Biosystems).

**Table 1:** Primers used for amplification and sequencing of the selected genes and *ompA gene*.

**Region**	**Primer name**	**Sequence (5´-3´)**	**Amplicon size (bp)**
CTLon_0054	CTLon_0054_F	ATGCTTCATCTATGTGATGT	768
	CTLon_0054_R	TTACTCCTGGGTAACGACAT	
CTLon_0087	CTLon_0087_F	GCTTTATGGAGTGAGTACACT	574
	CTLon_0087_R	CCTGAAGAAATGAAGAGTGCT	
CTLon_0243	CTLon_0243 _F	TCGAAACCAGTTCTTATCTCT	1,020
	CTLon_0243 _R	GAGATCATTCAAAGCGTCTGT	
CTLon_0301	CTLon_0301 _F	TGGAACCCTAATAAAGTAGT	1,600
	CTLon_0301 _R	TTGCCTTAGTAATTTCTCCT	
*ompA*	*ompA*_F	AACCAAGCCTTATGATCGACGGAAT	990
	*ompA*_R	CAATACCGCAAGATTTTCTAGATTTCA	

### Phylogenetic analysis

For phylogenetic reconstructions, the amplified concatenated nucleotide sequence for each gene included in each MLST scheme was used. The genes chosen for each scheme were aligned, concatenated, and edited using the ClustalW algorithm implemented in MEGA 7. A nucleotide substitution model for each gene was selected using the jModeltest1.0 software. Maximum likelihood (ML) phylogenetic trees were reconstructed using PhyML 3.0. The bootstrap test (1,000 replicates) was used to calculate branch support. Support values >90% were considered statistically significant. ML phylogenetic trees were constructed using MEGA 7 based on the general time reversible model. A consensus tree was generated using the TreeAnnotator software. To avoid introducing sequencing errors, mutations at the ends of the sequenced fragments were not considered. In our scheme (selected genes and *ompA*) the phylogenetic analysis was performed using 4,042 bp.

Transmission networks were constructed based on the maximum parsimony method using the reconstructed evolutionary tree and assuming that the L2b strain reported in Netherlands (2003) was the origin of the global epidemic.

## Results

### Diversity analysis and selected genes

**Figure 1:**
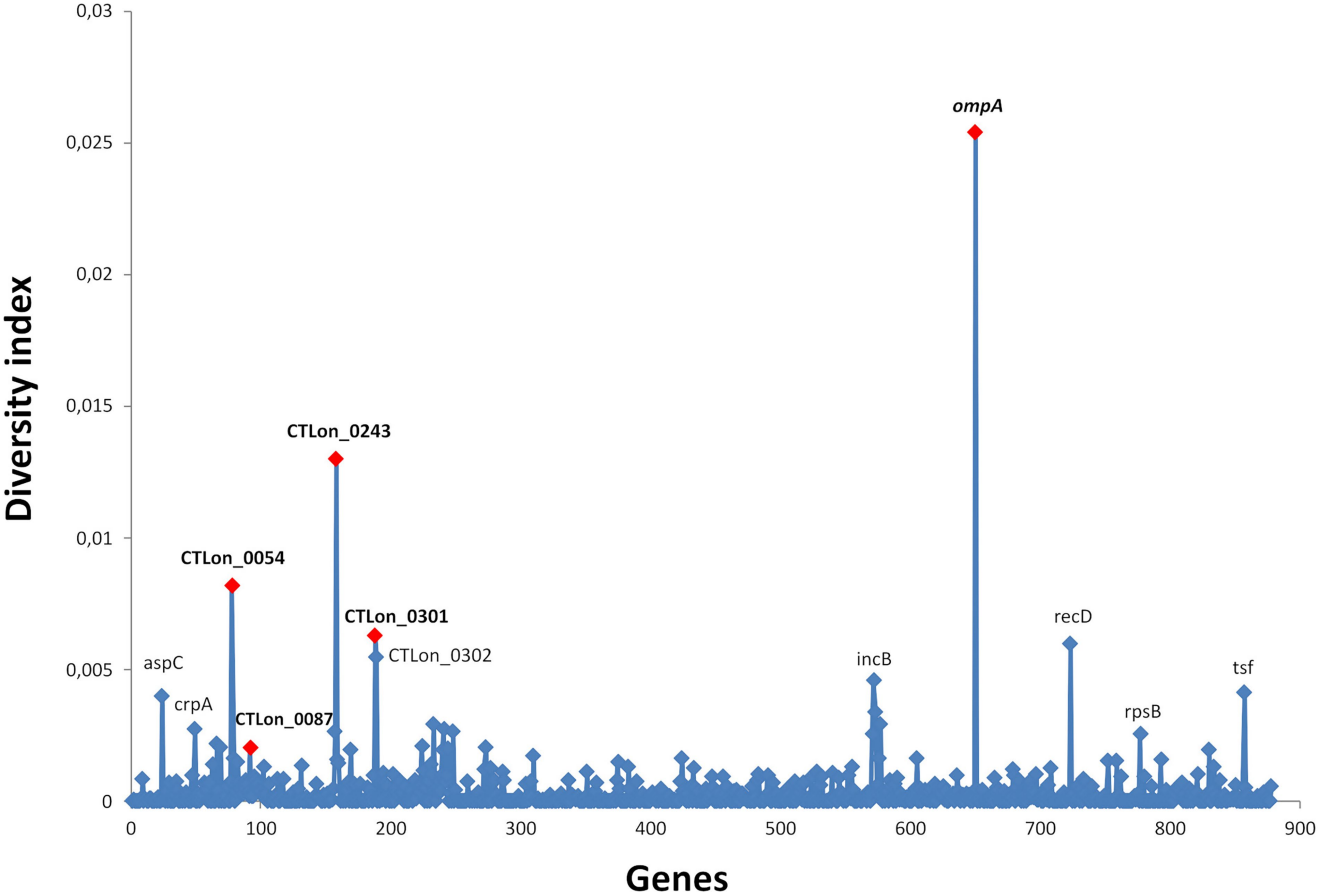
Mean evolutionary diversity (D index) of the core-genome of L-genotypes of *Chlamydia* *trachomatis*. A total of 878 genes were analyzed using the Tamura-Nei model. The selected genes for this study are marked in red.

The initial diversity analysis performed using the complete genome sequences available in public databases allowed the identification of the most variable genes (Figure 1). According to the D index obtained and using AM884177 as reference strain, the new genes selected were CTLon_0054 (ABC transporter ATP-binding protein), CTLon_0087 (conserved hypothetical protein), CTLon_0243 (putative membrane protein that contains the catalytic C-terminal domain of peptidase_C48 superfamily) and CTLon_0301 (conserved hypothetical protein), used in combination with *ompA*. Although the D index of CTlon_0087 was lower that of the other genes, this gene was still selected alongside the other genes, as they were the only genes capable of discriminating the recombinant L2c (analysis not shown). The D indices for each molecular marker included in the proposed scheme and the 12 molecular markers of the two previously described CT MLST schemes are shown in Table 2. Based on the complete genomes, the scheme of Dean et al. (1991) is not suitable for the molecular characterization of LGV epidemiology because it does not have sufficient discriminatory power among L-genotypes, and consequently, it was excluded in subsequent analyses. For the scheme proposed by Pederesen et al., CT1299 and CT1335 were identical in all L-genotypes; however, using CT1291, we identified three patterns (7C, 8C and 9C in the VNTR). However, this scheme was not used in the successive analyses because only one fragment (excluding *ompA*) showed some variability among the L-genotypes. In contrast, all the genes from the scheme proposed by Klint et al. (2007) showed variability among the L-genotypes. Therefore, the genes belonging to this scheme and our proposed scheme were sequenced in a selection of samples in the subsequent experiments.

**Table 2:** Mean evolutionary diversity (D index) for each marker of the previous MLST schemes and the one proposed in this study.

MLST scheme	Molecular marker	D index in L-genotypes
Dean et al. (2009)	*glyA*	0
	*leuS*	0
	*lysS*	0
	*mdhC*	0
	*pdhA*	0
	*pykF*	0
	*yhbG*	0
Klint et al. (2007)	*hctB*	0.001
	CT058	0.001
	CT144	0.007*
	CT172	0.001
	*pbpB*	0.001
This study	CTLon_0054	0.008
	CTLon_0087	0.002
	CTLon_0243	0.013
	CTLon_0301	0.006
	*ompA*	0.025

These values were obtained based on 29 available whole-genome sequences. The mean distance across the chlamydial genome was estimated in 0.0003 (SD ± 0.0012) with a 95% confident interval (95%CI) of 0–0.0026. According to the data showed in this table, all selected genes are into normal distribution, except *ompA*.

* The molecular marker CT144 from the scheme proposed by Klint et al. (2007) was not included in our scheme because D = 0 when the analysis was performed using L2 sequences.

### Comparison between the different strategies for the resolution by phylogenetic reconstruction

**Figure 2:**
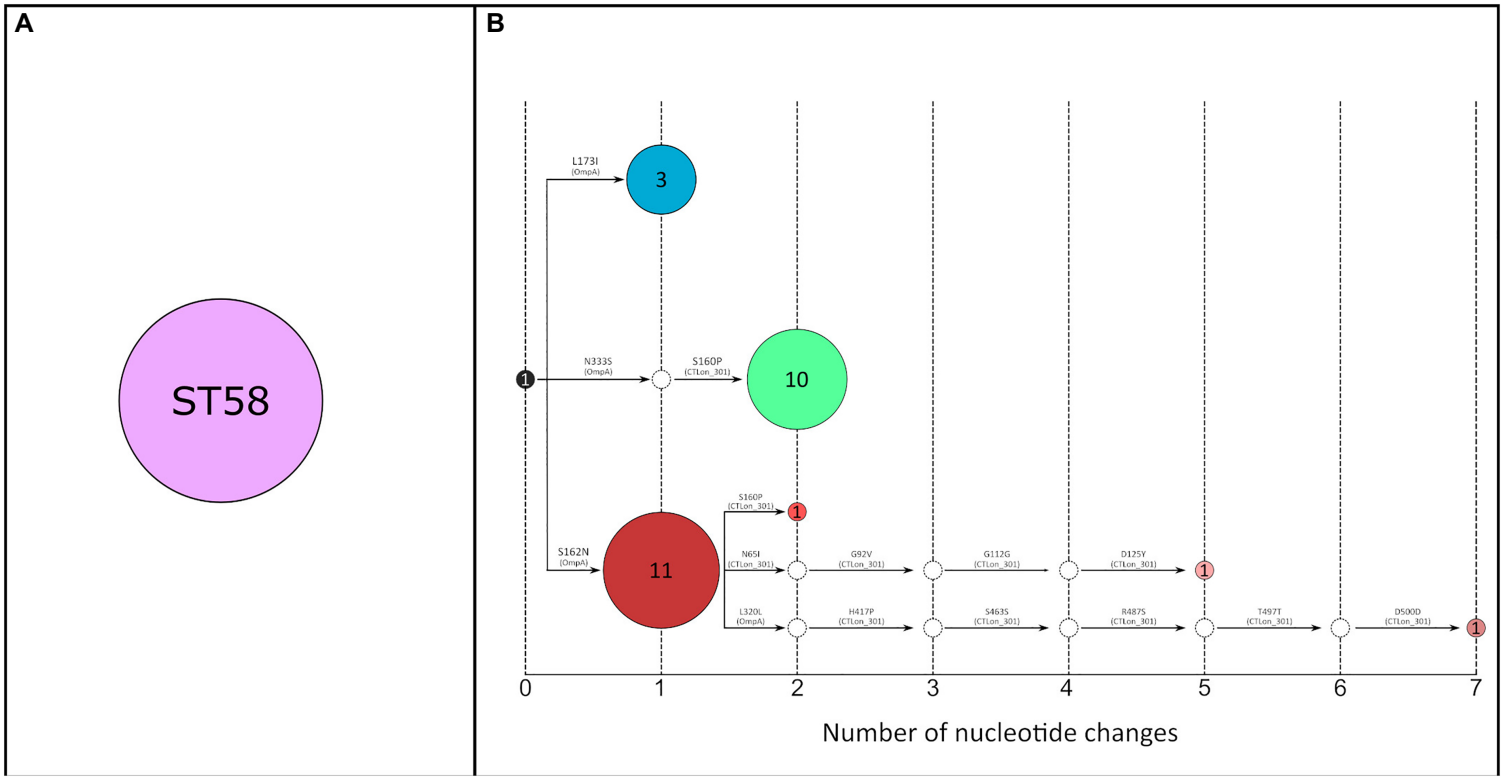
**(A)** Transmission network constructed using the concatenated genes of the MLST scheme proposed by Klint et al. (2007) (2,682 bp) (*n* = 28). All samples belong to ST58 (purple circle). **(B)** Transmission network constructed using the concatenated genes of the scheme proposed in this study (4,042 bp) (*n* = 28). A total of seven variants were identified. The number inside the circles corresponds to the number of samples included in each node. Each color represents a different variant. White circles correspond to hypothetical ancestors; the black circle corresponds to the L2b variant reported in Netherlands; red-tone circles correspond to L2b-*ompA* L2 variants; blue-tone circles correspond to L2bV1 variants and green-tone circles correspond to L2bV7 variants. Amino acid changes are indicated in each arm referred to as L2b/UCH-1 (AM884177), and the gene where each mutation is located is shown in parentheses.

Three ML trees were constructed using the concatenated sequences of the selected genes from 28 clinical samples used in the scheme proposed by Klint et al. (2007) (Supplementary Figure S1A), the scheme proposed in this study (Supplementary Figure S1B), and the whole genomes available in GenBank, which was considered a reference strategy because of its highest resolution (Supplementary Figure S1C). The three approaches discriminated the main lineages defined by Harris et al. (2012), and a new lineage from South Africa (assembly 7054_3#87 and 7054_3#88). Using the scheme proposed in this study and the scheme proposed by Klint et al. (2007), all our samples were assigned to the L2b genotype. While using the scheme described by Klint et al. (2007), all sequences were allocated to a single clone (ST58). However, our proposal identified seven variants distributed in three main branches (Figure 2), corresponding to L2bV1, L2bV7, and L2 variants (according to the *ompA* sequence).

**Figure 3:**
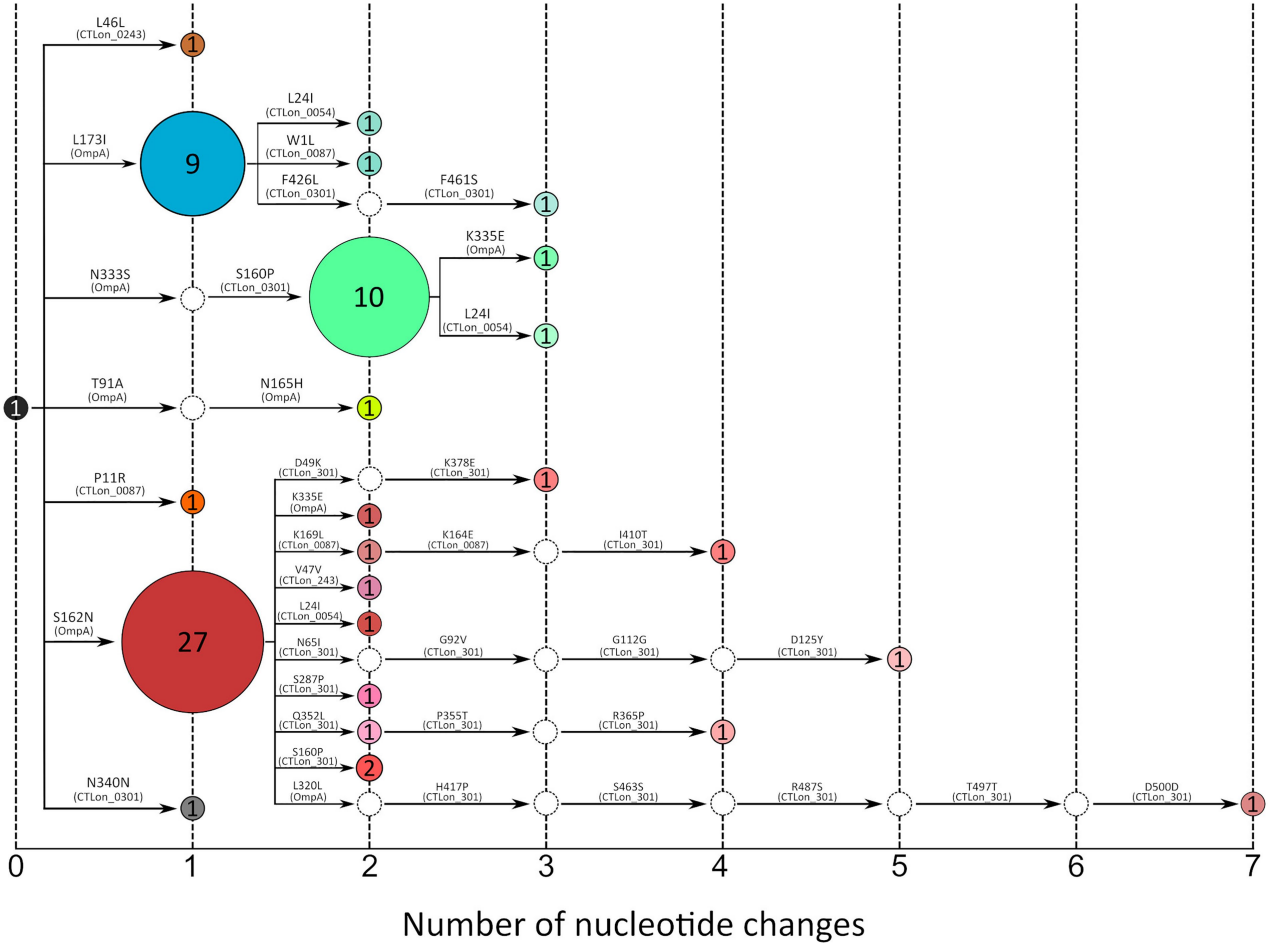
Transmission network constructed using the concatenated genes of the scheme proposed in this study (4,042 bp) (*n* = 68). The number inside the circles corresponds to the number of samples included in each node. Each color represents a different variant. White circles correspond to hypothetical ancestors; the black circle corresponds to the L2b variant reported in Netherlands; red-tone circles correspond to L2b-*ompA* L2 variants; blue-tone circles correspond to L2bV1 variants and green-tone circles correspond to L2bV7 variants. Amino acid changes are indicated in each arm referred to as L2b/UCH-1 (AM884177), and the gene where each mutation is located is shown in parentheses.

Once our scheme demonstrated higher discrimination capacity than previously described schemes using 28 samples, we obtained the concatenated sequence for 40 additional samples, resulting in a total of 68 samples (from 68 patients). An evolutionary reconstruction allowed us to determine the dispersion and diversification of variants in Madrid over 15 months (Figure 3). The analysis confirmed the presence of the three major transmission chains; however, a higher diversity of variants was observed evolving from the most ancestral L2b variant related to Netherlands and the three main variants involved in the respective transmission chains. One of the chains was composed of 40 patients, of whom 27 were infected by the founder variant (L2b-*ompA* L2); the second chain was composed of 12 individuals, of whom 10 were infected by the founder variant (L2bV7); and the third chain was composed of 12 patients, of whom 9 were infected with to the founder variant (L2bV1).

### Molecular characterization of lymphogranuloma venereum epidemic in Madrid

**Figure 4:**
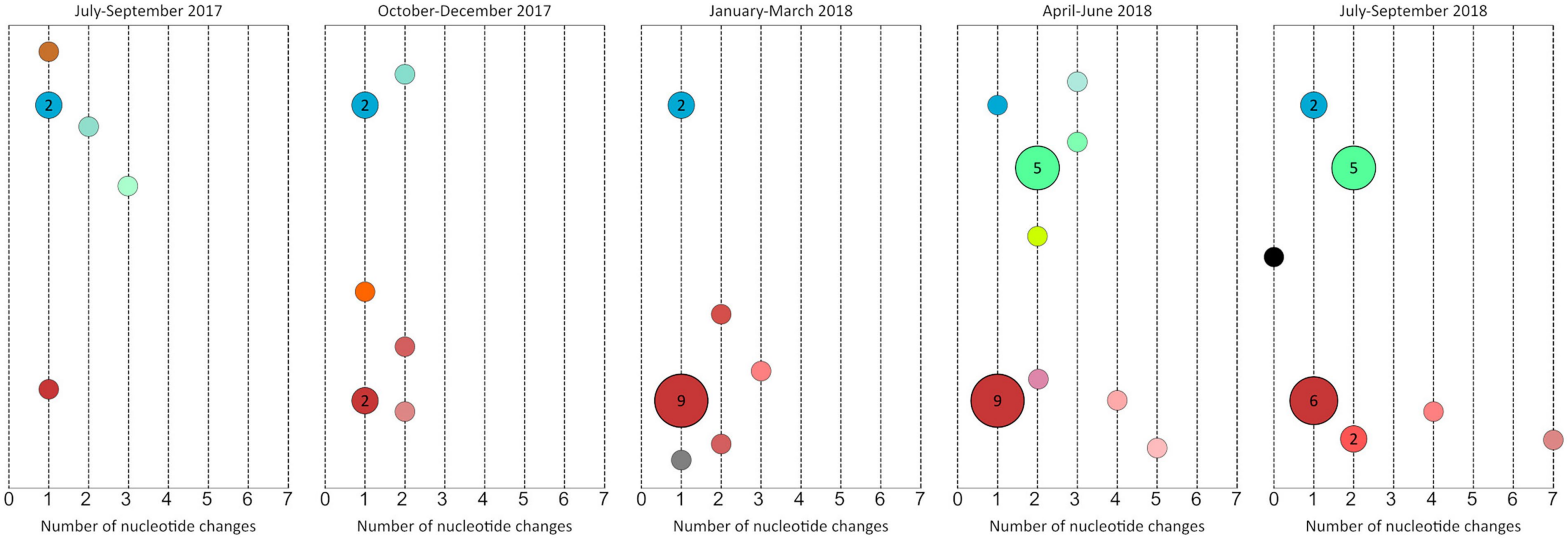
Quarterly representation of the variants detected using the concatenated genes of the scheme proposed in this study (4,042 bp) from July 2017 to September 2018 (*n* = 68). The number inside the circles corresponds to the number of samples included in each node. If no number is represented, it corresponds to one sample. The black circle corresponds to the L2b variant reported in Netherlands; red-tone circles correspond to L2b-*ompA* L2 variants; blue-tone circles correspond to L2bV1 variants and green-tone circles correspond to L2bV7 variants. The x-axis represents the number of nucleotide changes in each variant with respect to L2b/UCH-1 (AM884177).

To better understand the spread of the variants identified in the study period, temporal and spatial evolution were analyzed. The detected L-genotypes were represented quarterly, following the proposed scheme in the previous section (Figure 4). The L2b-*ompA* L2 variant increased from the third trimester and since then this cluster has been progressively diversifying. L2bV7 also increased from the fourth trimester, whereas L2bV1 was maintained during the entire study period, but with a low transmission level. Notably, in the last trimester, we detected an L2b identical to the original L2b identified in Amsterdam in 2003 (AM884177).

**Figure 5:**
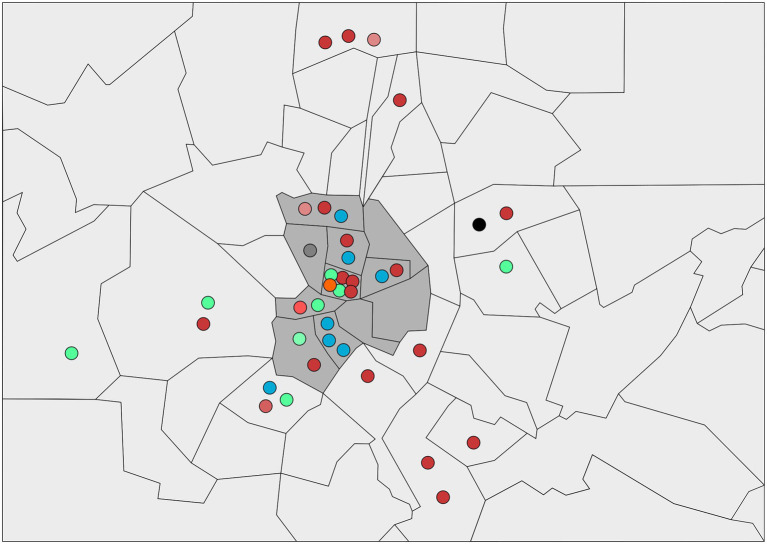
Schematic representation of the districts of Madrid where LGV cases were identified. The different circles correspond to the L2b variants detected in the study. Each color represents a different variant. The black circle corresponds to the L2b variant reported in Netherlands; red-tone circles correspond to L2b-*ompA* L2 variants; blue-tone circles correspond to L2bV1 variants and green-tone circles correspond to L2bV7 variants. The central district is identified in grey background.

Based on the spatial distribution of cases detected according to the postal codes (Figure 5), the L2b-*ompA* L2 variant and its evolved variants were detected around many districts in the metropolitan area. In contrast, L2bV1 was detected in five postal codes in the central and southwestern areas, whereas L2bV7 variants were detected in seven postal codes in the central and western areas. In the central districts, all variants were identified, whereas in the peripheral districts, the diversity of the variants decreased.

## Discussion

Since the 1960s, genotyping of CT has been based on characterizing *ompA*, which has been successfully used for the typing of this organism, but with a low discriminatory power from an epidemiological perspective. MLST schemes based on the sequencing of housekeeping genes are classically used in molecular epidemiology studies for different pathogens and may be useful in cases where the ultimate resolution of the WGS is not easy to implement, such as in non-cultivable or non-easily cultivable microorganisms, such as CT. WGS is the strategy with the most discriminatory power; however, the moderate success and technical difficulties of this strategy for this microorganism are hindering its generalization in many clinical laboratories. Therefore, tree different MLST schemes for *Chlamydia* have been proposed (Klint et al., 2007; Pannekoek et al., 2008; Dean et al., 2009) and are frequently used. However, their application to L-genotypes is limited. For example, the MLST of Pannekoek et al. (2008) grouped all L-genotypes into an identical MLST type. The scheme described by Dean et al. (2009). only discriminates L2b from other L-genotypes. The study of Klint et al. (2007), proposed for the first time the use of highly polymorphic genetic regions for typing instead of the conserved household genes (Klint et al., 2007) based on all CT genotypes. However, previous studies, including samples from different parts of the world (Christerson et al., 2010; Isaksson et al., 2017; Levy et al., 2018), confirm that this model does not have enough discriminatory power for LGV isolates. Recently, the MLVA-*ompA* strategy (Pedersen et al., 2008) developed for CT (Labiran et al., 2016), was used in the LGV epidemic (Manning et al., 2021), identifying five different variants among 230 samples collected over 17 months.

To address this limitation, we proposed a new combination of genes with high diversity among L-genotypes. Using diversity analysis of the WGS available, we selected four genes with high diversity among L-genotypes (CTLon_0054, CTLon_0087, CTLon_0243 and CTLon_0301) for use in combination with *ompA*. The resolution of our scheme was compared with the scheme proposed by Klint et al. (2007), using 28 patient samples. Although this was the only MLST scheme whose genes showed diversity among L-genotypes, all samples were classified as ST58, and the concatenated sequence did not reveal any differentiation (Supplementary Figure S1A). By contrast, using the same samples, our scheme indicated the existence of seven variants (Figure 2), revealing improved discriminatory power. This discriminatory power was used to analyze the LGV epidemiology in a short period, for which the sample size was extended to a total of 68 samples. The analysis revealed 24 different variants, most of which were grouped into three main nodes. These results are better than those of the MLVA-*ompA* employed for LGV epidemic in UK (Manning et al., 2021), yielding lower discrimination power.

Before molecular typing methods became available, transmission network analysis of STIs was performed using contact tracing. Owing to the availability of high-resolution typing techniques such as those proposed in this study and with an epidemiological survey, the sexual networks might be unveiled with higher precision. Moreover, schemes with great discrimination power offer the possibility of differentiating between reinfection and persistence (de Vries et al., 2015), which are clinical situations that have caused controversy in the management of the patients and the risk of treatment failure.

In contrast, the spatio-temporal distribution of L-variants identified in this study could help increase our perception of the endemic situation in Madrid. The temporal detection of L-variants provided new information. For instance, the progressive diversification L2b-*ompA* L2 revealed a very active transmission chain, or the detection of L2b identical to ancestral variant suggests re-introductions from other regions or low screening in high-risk populations. Moreover, the sudden increase in L2bV7 cases in April 2018 would suggest new outbreaks associated with sexual networks. The spatial distribution of L-variants revealed the radial spreading from the central district to the peripheral regions. Therefore, the central district was a crucial hotspot in the LGV endemic situation in Madrid.

The phylogenetic analysis of concatenated genes of Klint et al. (2007) scheme and our proposed genes revealed that all variants belonged to the L2b lineage, even though some of them have an *ompA* other than L2b. Moreover, the whole-genome analysis of L-genotypes revealed that all variants belonged to L2b regardless of *ompA* (Seth-Smith et al., 2021). Previous studies reported that *ompA* or parts of *ompA* may be exchanged between CT strains by recombination (Harris et al., 2012; Labiran et al., 2012). However, a recent study on the diversification of L-variants revealed that the single nucleotide polymorphisms (SNPs) present in the LGV lineage arose by mutation (Seth-Smith et al., 2021).

Our study has several limitations. First, the low number of sequenced samples did not allow us to obtain a more complete view of the LGV epidemic. Moreover, the availability of whole genomes sequences was limited, and the final diversity analysis was performed using L2 sequences, as only one whole-genome sequence from L1 and L3 was available.

In summary, although WGS (whole genome sequencing) is the best tool for improving the understanding of phylogenetic relationships, it currently requires a more complex infrastructure and analysis with higher costs. Although culture-independent methods are available (Taylor-Brown et al., 2018) for CT, their application remains difficult. Our scheme has proven to be useful for appropriate discrimination of LGV strains, revealing a more complex distribution of LGV than previously thought, with different variants circulating in high-risk populations. Moreover, this method can be used for a detailed resolution of local diversity in a short period, monitoring therapy and contact tracing.

## Data availability statement

The sequences obtained in this study were deposited in GenBank under the following accession numbers: OP611811 to OP611878 (*ompA*), OP611879 to OP611946 (CTLon_0054), OP611947 to OP612014 (CTLon_0087), OP612015 to OP612082 (CTLon_0243) and OP612083 to OP612150 (CTLon_0301).

## Author contributions

JG-A and JG: conceptualization. LM-G, MR-D, JG-A, and JG: methodology. LM-G, JG-A, and JG: formal analysis and writing—original draft. LM-G, MR-J, and MR-D: experimental investigation. EO, TP, AC, and MS-C: clinical investigation. LM-G, JG-A, TP, AC, MR-J, EO, MS-C, MR-D, RC, and JG: writing—review and editing. All authors contributed to the article and approved the submitted version.

## Funding

This study was supported by Instituto de Salud Carlos III (ISCIII), PI16/01242, PI20/01397, co-funded by the European Union; and also supported by the Spanish Network CIBERESP (CB06/02/0053).

## Conflict of interest

The authors declare that the research was conducted in the absence of any commercial or financial relationships that could be construed as a potential conflict of interest.

## Publisher’s note

All claims expressed in this article are solely those of the authors and do not necessarily represent those of their affiliated organizations, or those of the publisher, the editors and the reviewers. Any product that may be evaluated in this article, or claim that may be made by its manufacturer, is not guaranteed or endorsed by the publisher.

## Footnotes

^1^ ftp.ncbi.nlm.nih.gov/genomes/genbank/bacteria/?/latest_assembly_versions
