# Supplementary figures and images for: Specific high-resolution scheme to improve understanding of the spatio-temporal dispersion of lymphogranuloma venereum epidemic
Source: Front Microbiol. 2022 Dec 20;13:1056216. doi: 10.3389/fmicb.2022.1056216 (PMC9808035; doi:10.3389/fmicb.2022.1056216)

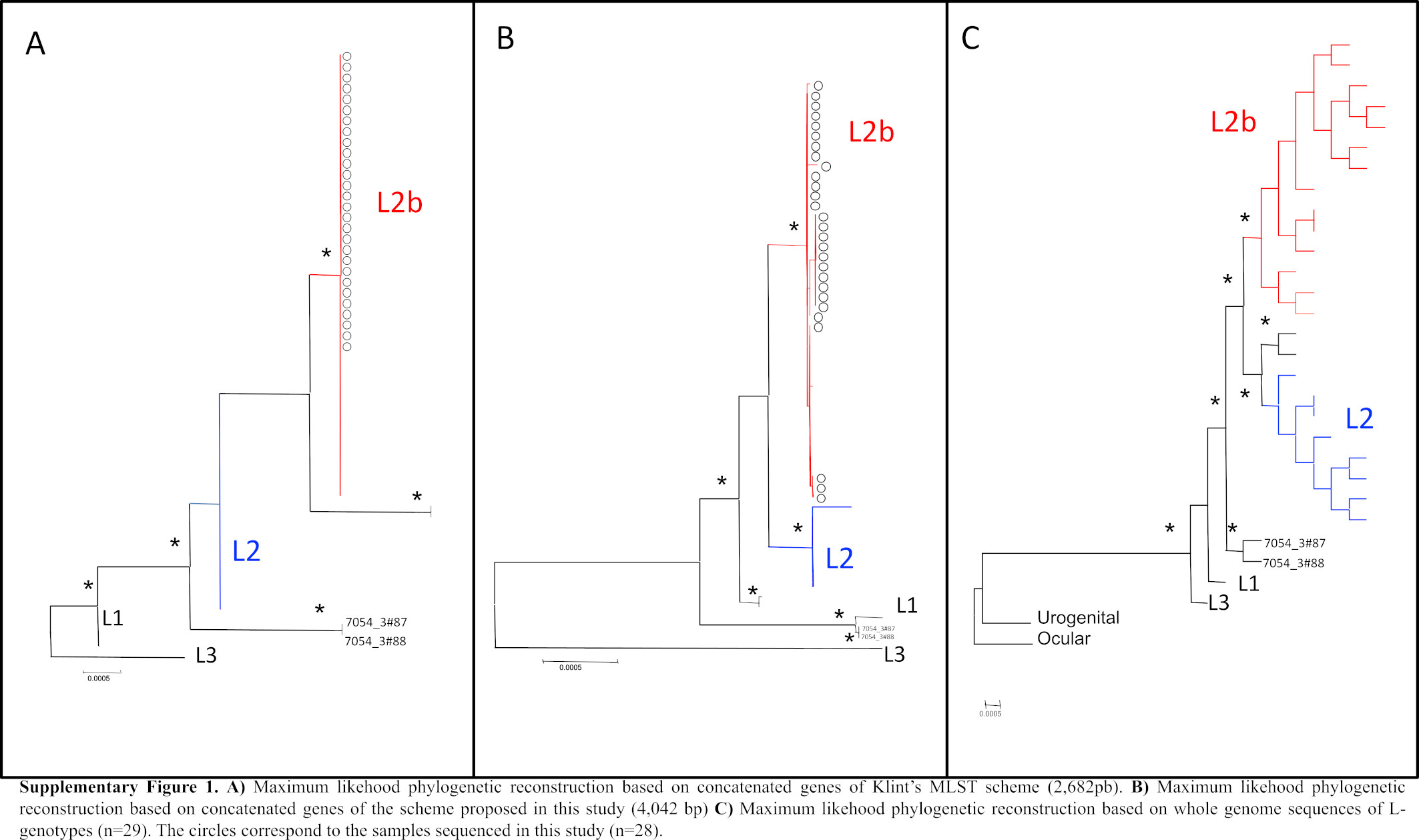

Supplement: Supplementary file 1 [file Image_1.JPEG]
